# Carboxymethyl Cellulose Hydrogel from Biomass Waste of Oil Palm Empty Fruit Bunch Using Calcium Chloride as Crosslinking Agent

**DOI:** 10.3390/polym13234056

**Published:** 2021-11-23

**Authors:** Nur Fattima’ Al-Zahara’ Tuan Mohamood, Abdul Hakam Abdul Halim, Norhazlin Zainuddin

**Affiliations:** Department of Chemistry, Faculty of Science, Universiti Putra Malaysia, Serdang 43400, Malaysia; fatimazahara.tm@gmail.com (N.F.A.-Z.T.M.); hakam5996@gmail.com (A.H.A.H.)

**Keywords:** oil palm biomass waste, anionic hydrogel, swelling, carboxymethyl cellulose, salt crosslinking agent

## Abstract

Carboxymethyl cellulose (CMC) is modified cellulose extracted from oil palm empty fruit bunch (OPEFB) biomass waste that has been prepared through etherification using sodium monochloroacetate (SMCA) in the presence of sodium hydroxide. In this research, CMC hydrogel was prepared using calcium chloride (CaCl_2_) as the chemical crosslinker. Throughout the optimization process, four important parameters were studied, which were: (1) CMC concentration, (2) CaCl_2_ concentration, (3) reaction time, and (4) reaction temperature. From the results, the best gel content obtained was 28.11% at 20% (*w*/*v*) of CMC with 1% (*w*/*v*) of CaCl_2_ in 24 h reaction at room temperature. Meanwhile, the degree of swelling for CMC hydrogel was 47.34 g/g. All samples were characterized using FT-IR, XRD, TGA, and FESEM to study and compare modification on the OPEFB cellulose. The FT-IR spectrum of CMC hydrogel showed a shift of COO^−^ peaks at 1585 cm^−1^ and 1413 cm^−1^, indicating the substitution of Ca^2+^ into the CMC molecular chains. The XRD diffractogram of CMC hydrogel showed no observation of sharp peaks, which signified an amorphous hydrogel phase. The CrI value also proved the decrement of the crystalline nature of CMC hydrogel. TGA–DTG thermograms showed that the T_max_ of CMC hydrogel at 293.33 °C is slightly better in thermal stability compared to CMC. Meanwhile, the FESEM micrograph of CMC hydrogel showed interconnected pores indicating the crosslinkages in CMC hydrogel. CMC hydrogel was successfully synthesized using CaCl_2_ as a crosslinking agent, and its swelling ability can be used in various applications such as drug delivery systems, industrial effluent, food additives, heavy metal removal, and many more.

## 1. Introduction

Polysaccharides are polymeric and complex carbohydrates that are generated as repeating units of monosaccharides, the simplest carbohydrates, and are linked by glycosidic linkages. Polysaccharides make up the majority of biomass and are thought to account for more than 90% of the carbohydrate material in nature. This natural polymer is formed either linearly with a straight chain of monosaccharides or branched with arms contingent on the monosaccharide link and the location of the carbon to which it is attached [1]. Polysaccharides, particularly cellulose, have a linear to highly branched structure, with main walls composed of cellulose, hemicellulose, lignin, and pectin. Various chemical processes have been employed to change existing polysaccharide structures throughout the years to improve and tailor their features [2].

Cellulose, the most prevalent polymer on earth, gives plants strength and is a bio-renewable ecologically benign raw resource [3]. Cellulose is made up of two anhydroglucose units (AGU) that are joined together by β-1,4-glycosidic bonds [4]. Three hydroxyl groups of AGU play a significant part in leading the crystallinity of the polymer due to their capacity to create hydrogen bonds. Because of its high crystallinity and rigidity as a result of its long and linear chains with intermolecular hydrogen bonding, cellulose is insoluble in water and most organic solvents [5]. To overcome this critical issue and improve the water insolubility disadvantage that restricts cellulose’s adaptability in numerous industries and applications, cellulose must undergo chemical modification such as esterification or etherification. The modification of cellulose is crucial in order to enhance its industrial demands, whereby alterations can result in a wide range of cellulose utilizations. The conversion of cellulose into carboxymethyl cellulose (CMC), hydroxyethyl cellulose (HEC), hydroxyethylmethyl cellulose (HEMC), cationic cellulose (CC), and a few more would undoubtedly boost the value of native cellulose because these cellulose derivatives are widely used in a range of industrial applications such as food additives, detergents, agriculture, pharmaceuticals, and many others. The alteration of cellulose into CMC will enhance its physicochemical properties such as biocompatibility, biodegradability, swelling power, and water solubility.

Malaysia, as one of the world’s major oil palm producers, increased its planted area from 5.74 million hectares in 2016 to 5.81 million hectares in 2017 [6]. Biomass waste, particularly from the oil palm empty fruit bunch (OPEFB), has generated more than 18,000 tonnes and cellulose extracted from OPEFB has been determined to be 93% pure [7]. Since the purity of cellulose derived from OPEFB is high, it may be converted into a promising and profitable material with enormous opportunities in a variety of applications. OPEFB biomass waste has low economic value and most of the time poses a disposal challenge. Conventionally, OPEFB waste is often burnt, disposed of in landfills, or composted to organic fertilizer [8,9]. Maximizing waste energy recovery is beneficial for both environmental and economic reasons. The modification of OPEFB cellulose into CMC is crucial for improving the functional characteristics and performance of biomass waste cellulose.

Further modification of CMC into CMC hydrogel will raise the profitability of this polymeric material. The term “hydrogel” refers to a three-dimensional polymeric network of hydrophilic chains that can shrink, swell, and absorb a large amount of water [10]. It swells well in an aqueous solution but stays insoluble due to chemical or physical crosslinking between individual polymeric chains [11]. The rate of water absorption is determined by the presence of functional groups [12], availability of hydrophilic groups [13], state of water [14], and crystallinity of cellulose [15] in the polymeric hydrogel. The swelling property of the hydrogel has been widely used in a variety of applications, and advantages for their usage include the ability to encapsulate biomacromolecules such as proteins and DNA due to the hydrophobic interaction [16]. Hydrogel can be considered a smart material because of its capability to swell in different media, changing the structure due to certain external responses such as temperature, pH, ions, and substance concentration [17]. Interestingly, hydrogel can be produced by multiple routes of alteration techniques. For instance, CMC-HEC aerogel was successfully developed using a supercritical CO_2_-assisted process. It was found that CMC-HEC aerogel can absorb water more than 500 times the original weight of the aerogel and is best used for agricultural applications [18], while CMC methacrylate hydrogel prepared from photopolymerization crosslinking efficiently showed better diffusivities of bovine serum albumin, which was studied as a controlled release device [19].

In this research, oil palm empty fruit bunch (OPEFB) cellulose is chosen due to its abundance availability from the oil palm milling process in Malaysia. Improper handling of this solid waste can contribute to environmental concerns, burdening industry operators with waste disposal difficulties and increasing operational costs. The main objectives of this research are to modify the OPEFB cellulose into CMC via carboxymethylation and convert it into CMC hydrogel via ionic crosslinking using CaCl_2_ with thorough optimization. Since CaCl_2_ has high solubility in an aqueous solution, the experiment was easy to handle. Up to date, there is no study reported on CMC hydrogel from OPEFB waste crosslinked with CaCl_2_, and therefore, this study can contribute to the exploration of hydrogel from OPEFB. 

## 2. Materials and Methods 

### 2.1. Materials

Cellulose of OPEFB was supplied by Biorefinery Complex, Universiti Putra Malaysia, Serdang, Selangor, Malaysia. Sodium monochloroacetate (SMCA), methanol (100%), ethanol (95%), sodium hydroxide (NaOH, 99%) pellet, glacial acetic acid (99.8%), and calcium chloride (CaCl_2_) were purchased from R&M Chemicals, Selangor, Malaysia, while isopropanol (IPA, 98%) was purchased from Sigma Aldrich, St. Louis, MO, USA. All chemicals used in this study are of analytical grade and used without further purification. Distilled water was used throughout the experiment. 

### 2.2. Preparation of CMC

In a 250 mL beaker, 5.0 g of OPEFB cellulose was added and then followed by 10 mL of 30% (*w*/*v*) NaOH solution dropwise. Then, 100 mL of IPA was added to the mixture and mechanically stirred for an hour at room temperature. To complete the carboxymethylation reaction, 6.0 g of SMCA was added and the reaction continued for three hours at 45 °C. Then, the mixture was filtered, and the slurry of CMC was soaked in 300 mL of absolute methanol overnight. CMC was neutralized with glacial acetic acid then sieved and washed with 70% ethanol, followed by 99.7% ethanol. The washing process was repeated three times, and CMC was oven-dried for 24 h at 60 °C. CMC was kept in an air-tight container.

### 2.3. Preparation of CMC Hydrogel

**Figure 1:**
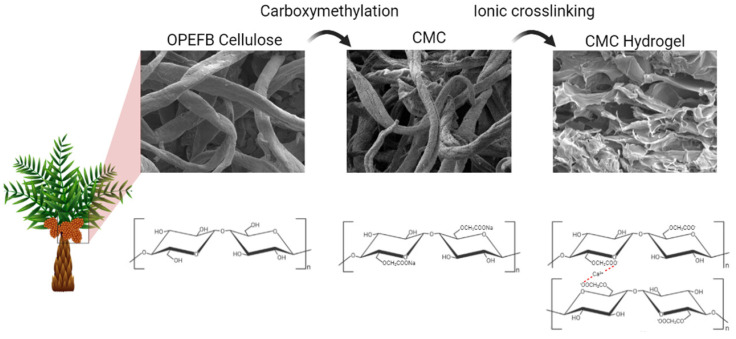
Diagrammatic scheme of conversion of OPEFB cellulose into CMC hydrogel.

CMC of 10–30% (*w*/*v*) was dissolved in 1–5% (*w*/*v*) of CaCl_2_ solution. The mixture was stirred homogeneously until a paste-like solution of CMC–CaCl_2_ was obtained and placed in a Petri dish. The CMC–CaCl_2_ paste was left to crosslink for 12–96 h at room temperature up to 60 °C. The optimization of the preparation of CMC hydrogel was carried out by modifying and controlling various parameters using the one-variable-at-a-time (OVAT) approach to reach the optimum percentage of gel content. Figure 1 shows the diagrammatic scheme of OPEFB cellulose carboxymethylation reaction and hydrogel ionic crosslinking.

### 2.4. Gel Content and Degree of Swelling of CMC Hydrogel

The gel content of CMC hydrogel was determined by measuring the insoluble part after the immersion of CMC hydrogel in distilled water for 72 h at room temperature. The distilled water was replaced every 24 h. The percentage of gel content was calculated using the following equation:

Percentage of gel content = (W_da_/W_db_) × 100% (1)

where W_da_ is the weight of dried hydrogel after immersion and W_db_ is the weight of hydrogel before immersion.

The degree of swelling of CMC hydrogel was carried out by the immersion of the CMC hydrogel in distilled water for 72 h at room temperature. The hydrogel was weighed after it reached equilibrium and the degree of swelling was calculated using the following equation:

Degree of swelling = (W_s_ − W_d_)/W_d_ (2)

where W_s_ is the weight of swollen hydrogel and W_d_ is the weight of the dried hydrogel.

### 2.5. Characterization

FT-IR spectroscopy is a technique used to determine the presence of functional groups in the CMC and CMC hydrogel. In this study, FT-IR was obtained from a Spectrum 100 Perkin-Elmer FT-IR spectrophotometer with a wavenumber of 400–4000 cm^−1^. The attenuated total reflection (ATR) sampling technique was used in conjunction with FT-IR spectroscopy and all samples were prepared in powder form.

The crystallinity of OPEFB cellulose, CMC, and CMC hydrogel was obtained from a Shimadzu XRD-6000 diffractometer with Cu Kα (λ = 1.5418 Å) radiation at room temperature. The machine was operated at 30 kV and 30 mA. The sample was placed in the aluminium sample holder and scattered intensity data were investigated in the scan range of 2–60° (2θ) with a continuous scanning rate of 2°/min. The crystallinity index (CrI) was calculated from the XRD diffractogram using the following equation [20] with the aid of OriginPro 2019 Graphing and Analysis software:

Crystallinity Index = (A_c_/A_c+a_) × 100% (3)

where A_c_ is the total area of crystalline peak and A_c+a_ is the total area of crystalline and amorphous peaks.

TGA of OPEFB cellulose, CMC, and CMC hydrogel was studied using TGA/SDTA-851 Mettler Toledo. It was used to determine the decomposition stage of samples and was performed under nitrogen flow and atmosphere air from 50 °C to 800 °C at the rate of 5 °C/min. The amount of weight loss as a function of temperature was examined and data were presented as a thermogram of weight loss versus temperature. 

FEI Nova NanoSEM 230 FESEM was used to study the surface morphology of OPEFB cellulose, CMC, and CMC hydrogel. Samples were sprinkled separately to the sample holder and were coated with gold before the analysis. A freeze-dried CMC hydrogel was used in this FESEM analysis.

In this study, the characterizations of the CMC hydrogel were performed using an optimized CMC hydrogel sample based on the optimization studies.

## 3. Results

### 3.1. Optimization of CMC Hydrogel

#### 3.1.1. Effect of CMC Concentration on CMC Hydrogel

**Figure 2:**
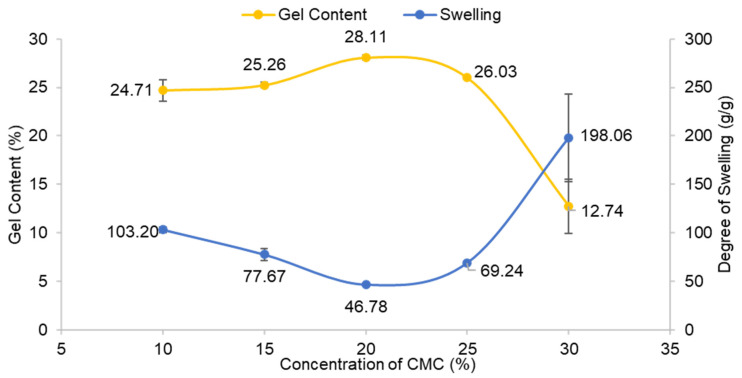
Effect of concentration of CMC on gel content and degree of swelling of CMC hydrogel. Error bar indicates the standard deviation of uncertainty.

**Figure 3:**
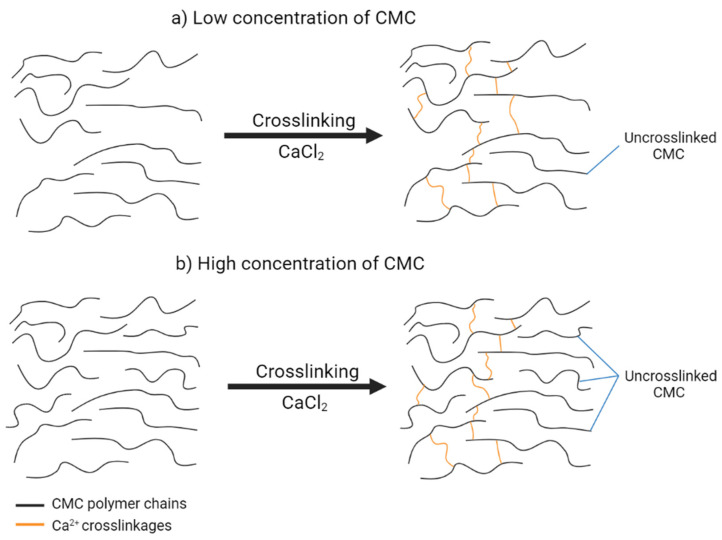
Schematic illustration of crosslinking reaction between CMC and CaCl_2_ at (**a**) low concentration of CMC and (**b**) high concentration of CMC.

Figure 2 depicts a graph of the gel content and degree of swelling of CMC hydrogel at various CMC concentrations. The controlled variables for this parameter were the concentration of CaCl_2_ at 1% (*w*/*v*) and 24 h reaction time at room temperature (27 °C). The gel content of CMC hydrogel steadily rose with CMC concentration starting at 10% (*w*/*v*) and reached a maximum of 20% (*w*/*v*) with 28.11%. It was found that at 25% (*w*/*v*) of CMC concentration and above, the percentage of gel content decreased. The increase in gel content up to 20% (*w*/*v*) of CMC may be due to higher crosslinkages between CMC chains. At high concentrations, CMC molecules are packed closer together, allowing Ca^2+^ ions to form linkages between the polymer chains. This finding is similar to the study by Fei et al. [16], who obtained a high gel fraction of hydrogel when the concentration of the CMC was from 5–30% only. However, as a higher concentration of CMC was used with a fixed amount of crosslinking agent throughout the experiment, the possibility for Ca^2+^ to create crosslinkages with CMC in the reaction was insufficient and limited, and thus gel content decreased. The schematic illustration of the crosslinking reaction between CMC and CaCl_2_ is shown in Figure 3. In this study, 20% (*w*/*v*) was chosen as the optimal concentration for this reaction. The swelling of CMC hydrogel is inversely proportional to its gel content. The degree of swelling was the lowest at the greatest gel content, which was 46.78 g/g. The degree of swelling dropped from 10% to 20% (*w*/*v*) of CMC and then began to rise at 25% (*w*/*v*) and higher CMC concentrations. At a high gel content of CMC, a high number of crosslinkages occurred in the CMC hydrogel network. As a result, water diffusion into the hydrogel voids became more challenging, and thus the swelling decreased. On the other hand, as the gel content decreased at a high concentration of CMC, the increase in the swelling implies a weak hydrogel due to low gel content and more water trapped inside the pores of the CMC hydrogel.

#### 3.1.2. Effect of CaCl_2_ Concentration on CMC Hydrogel

**Figure 4:**
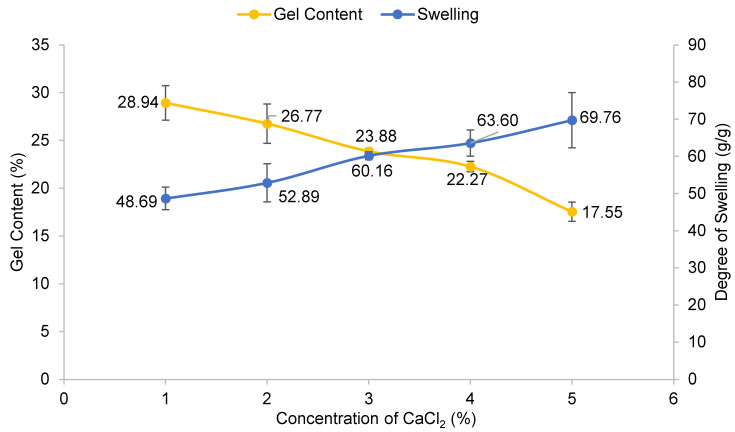
Effect of concentration of CaCl_2_ on gel content and degree of swelling of CMC hydrogel. Error bar indicates the standard deviation of uncertainty.

**Figure 5:**
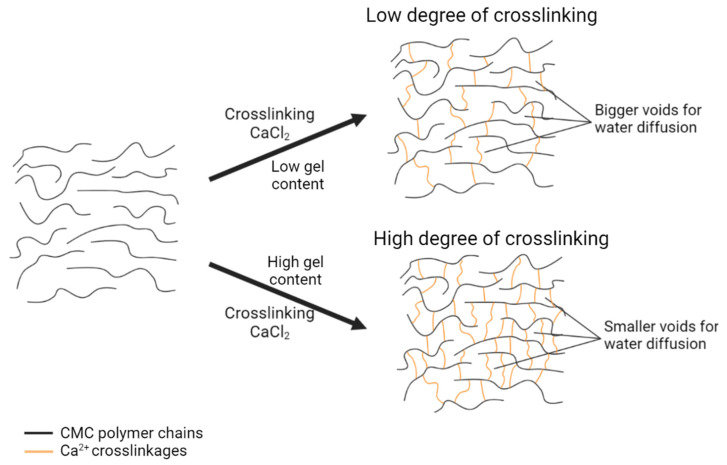
Schematic illustration of CMC–CaCl_2_ hydrogel with different degrees of crosslinking.

The concentration of CaCl_2_ varied from 1–5% (*w*/*v*), as illustrated in Figure 4. The controlled variables were 20% (*w*/*v*) CMC with 24 h reaction time and the experiment was carried out at room temperature. The highest percentage of gel content was 28.94% at 1% (*w*/*v*) CaCl_2_ and it gradually decreased at higher concentrations of CaCl_2_ solution. Sultana and Islam [21] published a similar discovery, stating that the ionic crosslinking of polymers increased as the electrostatic interaction between the ionic charges of polymer chains and multivalent cation Ca^2+^ increased. However, in this study, the gel content decreased as CaCl_2_ concentration increased. Reversible reactions may occur at greater CaCl_2_ concentrations, lowering the percentage of gel content. This finding is similar to the results of Che Nan et al. [22], which concluded that CMC chains lose their flexibility in high concentrations of CaCl_2_ and thus decrease in hydrodynamic molecular size and make the polymer chains agglomerate. Furthermore, CMC dissolution in water would be inhibited since CMC could not be fully hydrated in water with a greater CaCl_2_ concentration [23]. A similar trend in the swelling of CMC hydrogel where it is inversely proportional to the gel content was observed. As the gel content decreased, a lower degree of crosslinking occurred. Consequently, water molecules bound more easily to the surface of the hydrogel and diffused into the accessible voids, triggering hydrogel expansion owing to the water uptake of swelling response, as shown in Figure 5. According to Tolinski [24], the amount of interconnected polymer chains or the density of the gel component depends on the degree of crosslinking. The previous statement is supported by Maitra and Shukla [25], who stated that the swelling properties of hydrogel and transport of molecules are influenced by the degree of crosslinking in the polymeric chains of hydrogel. 

#### 3.1.3. Effect of Different Reaction Times on CMC Hydrogel

**Figure 6:**
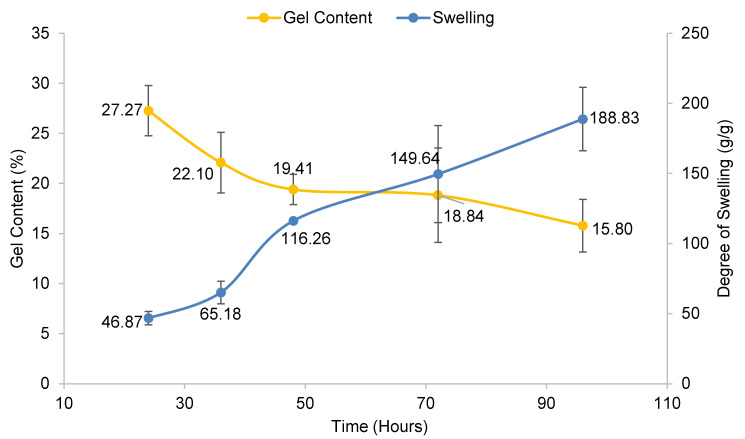
Effect of reaction time on gel content and degree of swelling of CMC hydrogel. Error bar indicates the standard deviation of uncertainty.

The influence of reaction time was studied, as shown in Figure 6. The reaction time varied from 24 to 96 reaction hours, and the controlled variables for this parameter were 20% (*w*/*v*) of CMC and 1% (*w*/*v*) CaCl_2_ performed at room temperature. The highest and best reaction time was at 24 h, which gave 27.27% of gel content. The trend displayed a gradual decrement of gel content when reaction time was prolonged. This might be due to the presence of NaCl (Na^+^ from –CH_2_COONa combined with Cl^−^ ion) in the hydrogel, which reduced electrostatic attraction in the polymer chains when CaCl_2_ was added at a longer reaction time. The chemical reaction is shown in Equation (4), as reported by Che Nan et al. [22]. Meanwhile, for the degree of swelling of CMC hydrogel, it showed a similar trend to the previous parameters; the effect of CMC and CaCl_2_ concentration.

Cell–OCH_2_COO-Na^+^ + CaCl_2_ → Cell-OCH_2_COO-Ca^2 +^ − -OOCCH_2_O–Cell + NaCl (4)

#### 3.1.4. Effect of Different Reaction Temperatures on CMC Hydrogel

**Figure 7:**
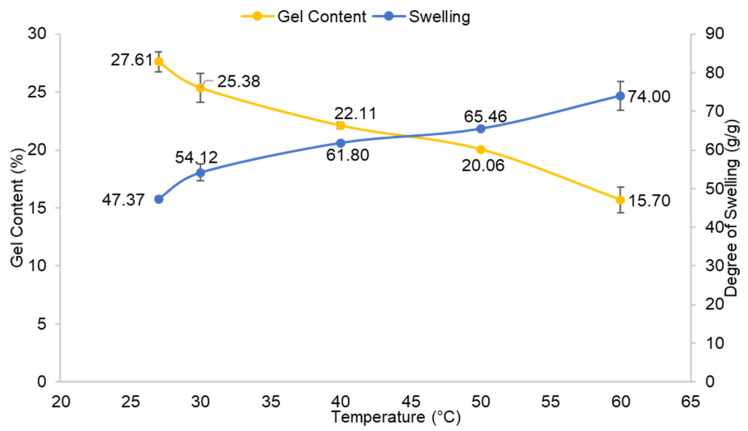
Effect of reaction temperature on gel content and degree of swelling of CMC hydrogel. Error bar indicates the standard deviation of uncertainty.

Figure 7 displays the percentage of gel content and degree of swelling of CMC hydrogel at various reaction temperatures. The constant variables were 20% (*w*/*v*) of CMC, 1% (*w*/*v*) CaCl_2_, and the reaction was completed at 24 h. This experiment was conducted at temperatures ranging from room temperature (27 °C) to 60 °C. The percentage of gel content of the CMC hydrogel dropped as the reaction temperature increased. It is found that room temperature gave the highest gel content of 27.61%. This might be due to the fact that the CMC hydrogel structure generated by CaCl_2_ ionic crosslinking is more stable at lower reaction temperatures [26]. As the temperature rose, a reversible reaction may have triggered the molecules, causing the hydrogel to disintegrate slowly. As a result, a low gel content of the CMC hydrogel was obtained. Conversely, Figure 7 indicates a consistent pattern to the proceeding factor for the degree of swelling of CMC hydrogel, which is inversely proportioned to the percentage of gel content.

### 3.2. Characterization

#### 3.2.1. Fourier Transform-Infrared Spectroscopy (FT-IR)

**Figure 8:**
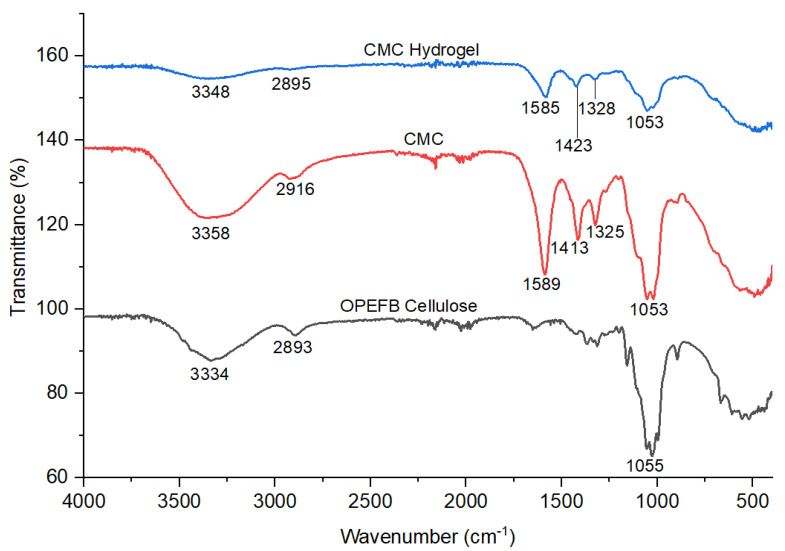
FT-IR spectra of OPEFB cellulose, CMC, and CMC hydrogel.

Figure 8 depicts the FT-IR spectrum of OPEFB cellulose with a strong absorption peak at 3334 cm^−1^, which is assigned to the hydroxyl group of the polysaccharide chain, –OH stretching, while the appearance of a shoulder peak at 2893 cm^−1^ is assigned to the –CH stretching vibration. These two peaks are the indication of the main polysaccharide nature where it is composed of glucose units. A significant stretching C–O vibration of primary alcohols and ethers in the cellulose backbone of the cellulose chain was observed at 1055 cm^−1^. According to Ngadi and Lani [27], the appearance of a peak at 1055 cm^−1^ might be attributed to the C–O–C pyranose ring stretching vibration and -CH stretching vibration at 3358 cm^−1^ and 2916 cm^−1^, respectively. Strong absorption peaks at 1589 cm^−1^ and 1413 cm^−1^ indicated the appearance of the asymmetrical and symmetrical stretching of the –COO group from –CH_2_COONa of SMCA with a strong alkali solution of NaOH. By comparing to the OPEFB cellulose, the existence of the additional peak at 1589 cm^−1^ in CMC has proved that CMC was successfully synthesized from the OPEFB cellulose via carboxymethylation reaction. Similar research by Tulain et al. [28] also reported that in the range of 1500–1700 cm^−1^ of the absorbance band, carboxyl groups and their salts can be observed, which allied with Na–CMC. The peak observed from the CMC spectrum at 1325 cm^−1^ is assigned to –OH bending vibration. The FT-IR spectrum of CMC hydrogel revealed the same –OH and –CH absorption bands at 3348 cm^−1^ and 2895 cm^−1^, respectively. However, these two bands are less intense compared to the OPEFB cellulose and CMC. The absorption peaks at 1585 cm^−1^ and 1423 cm^−1^ are attributed to the asymmetrical and symmetrical stretching of the –COO group. There is no significant difference in the FT-IR spectra of CMC and CMC hydrogel, where no new peak can be observed in CMC hydrogel. This could be due to the fact that crosslinking only involved the substitution of Na^+^ with Ca^2+^ from CaCl_2_ as the crosslinking agent. There was only slight shifting in the wavenumber after Ca^2+^ ionic crosslinking on the CMC chain from 1589 cm^−1^ to a lower wavenumber (1585 cm^−1^) and 1413 cm^−1^ to a higher wavenumber (1423 cm^−1^) due to the bidentate of the carboxylate group to Ca^2+^ [29]. In addition, both peaks showed less intensity after the ionic crosslinking reaction compared to the CMC, which reflects the decrement of the crystalline nature of the hydrogel [30]. The decrease in crystallinity of CMC hydrogel was proven by XRD analysis, which will be discussed in the next section.

#### 3.2.2. X-ray Diffraction (XRD)

**Figure 9:**
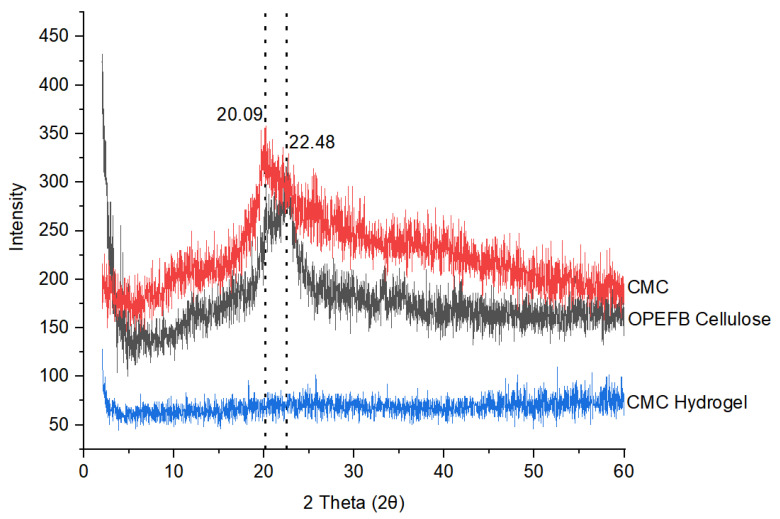
XRD diffractogram of OPEFB cellulose, CMC, and CMC hydrogel.

The XRD diffraction of OPEFB cellulose, CMC, and CMC hydrogel is plotted in Figure 9, while the crystallinity index (CrI) is tabulated in Table 1. CrI was used to study the change in structure and degree of crystallinity in OPEFB cellulose, CMC, and CMC hydrogel. 

The XRD pattern for OPEFB cellulose is shown at 2θ = 22° and CMC at 2θ = 20°, which corresponds to the amorphous region and small crystallites in the cellulose granules. Baharin et al. [31] reported that the diffraction peak 2θ = 22.6° of OPEFB cellulose is assigned to the crystalline phase, while Uyanga and Daoud [32] reported that commercial CMC with diffraction peak 2θ = 20.03° indicates the amorphous phase. The crystallinity index (CrI) of OPEFB cellulose was found to be the highest among all samples at 48.70%, which indicates that there is a more crystalline phase in OPEFB cellulose compared to CMC and CMC hydrogel. The diffraction pattern of CMC showed that CMC has lower crystallinity compared to OPEFB cellulose as there was a less intense peak observed with a broad pattern at 2θ = 20°. The loss of crystalline nature is probably due to the replacement of hydroxyl groups in the samples with the inclusion of strong alkaline, NaOH, and water in the crystallites during carboxymethylation [33]. This finding is in good agreement with TGA data, which show that CMC has a lower maximum decomposition temperature compared to OPEFB cellulose due to the loss of the crystalline phase. The crystallinity reduction of the CMC has also caused this modified cellulose to experience excellent solubility in water [34]. Table 1 shows the CrI for OPEFB cellulose, CMC, and CMC hydrogel. The CrI for OPEFB cellulose and CMC was 48.70% and 32.77%, respectively. For the XRD pattern of CMC hydrogel, there was no appearance of a sharp peak, thus it can be concluded that CMC hydrogel was in the amorphous phase. The substitution of Ca^2+^ into the CMC formed a highly crosslinked CMC and thus disturbed the well-ordered cellulose structure and reduced the mobility of the cellulose chains. The CrI of the CMC hydrogel also showed a significant reduction at only 4.24%. This indicates that CMC hydrogel fully transformed into amorphous nature after some alterations of carboxymethylation and ionic crosslinking.

#### 3.2.3. Thermogravimetry Analysis (TGA)

**Figure 10:**
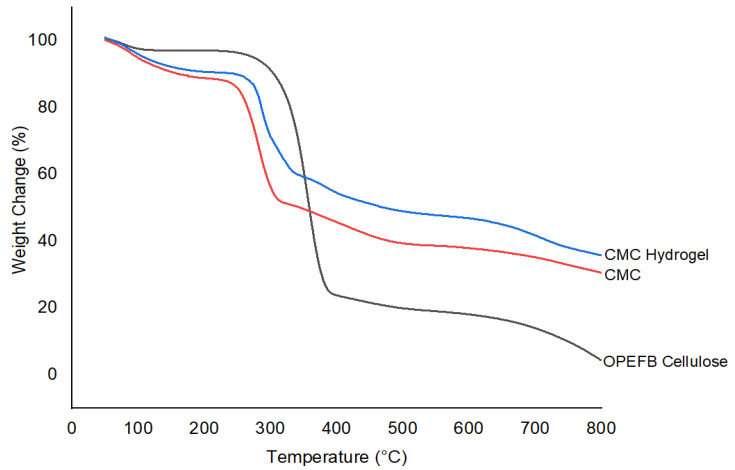
TGA thermograms of OPEFB cellulose, CMC, and CMC hydrogel.

**Figure 11:**
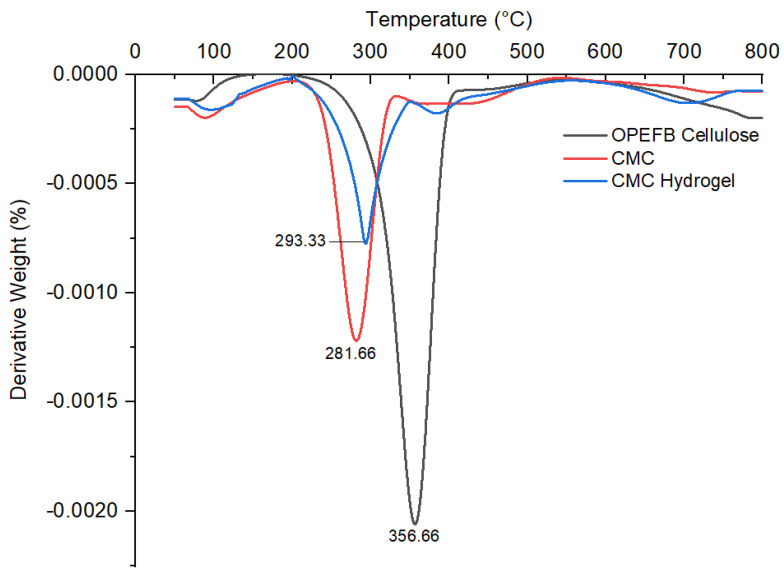
DTG thermograms of OPEFB cellulose, CMC, and CMC hydrogel.

TGA measures the amount and rate of change in a material’s weight in a monitored atmosphere as a function of temperature over time. It is mainly used to determine the material composition and predict thermal stability. In this study, all samples were analyzed at a temperature range of 50–800 °C for both TGA and DTG profiles, as shown in Figure 10 and Figure 11, respectively. The thermogram profiles showed that OPEFB cellulose, CMC, and CMC hydrogel decomposed at two stages. The first decomposition stage took place in the range of 50–180 °C, which indicated the removal of water loosely bound in the sample, and the second decomposition stage took place at around 200–550 °C, which was correlated to the degradation of the cellulose backbone.

The first decomposition stage for OPEFB cellulose, CMC, and CMC hydrogel was due to the loss of bound water, as well as the release of any volatile compounds from the surface and moisture entrapped inside the sample. The release of initial moisture is common in natural fiber. As depicted in TGA–DTG thermograms, small weight losses of OPEFB cellulose, CMC, and CMC hydrogel were observed at a temperature of 78.33 °C, 90.83 °C, and 96.66 °C, respectively. According to Mohtar et al. [35], any degradation or decomposition below 100 °C is not considered as a significant thermal event in the TGA study. The second decomposition stage was the most crucial stage for all three samples. The decomposition temperature of OPEFB cellulose started at 206.54 °C to 526.68 °C with a weight loss of 77.79% from its original weight, while CMC decomposed at the temperature range of 180.31–527.32 °C with a weight loss of more than half of the original weight after the modification of cellulose into CMC, which decomposed about 50.39%. For CMC hydrogel, after the ionic crosslinking reaction with CaCl_2_, the decomposition stage occurred at a slightly broad temperature at the range of 180.13–530.42 °C with 51.73% weight loss. The weight loss of OPEFB cellulose was the highest compared to CMC and CMC hydrogel because lignocellulosic materials are chemically active, which decompose thermo-chemically in the range of 200 °C to 500 °C, hence it is easier for the cellulosic materials to decompose at this temperature range. This result is in good agreement with the previous study by Khalid et al. [36]. For CMC, the decomposition at this second stage might be due to the degradation of the cellulose backbone and the removal of CO_2_ caused by decarboxylation [37]. Meanwhile, for CMC hydrogel, besides the elimination of CO_2_ molecules in the polymeric backbone of hydrogel, it was also most probably the degradation of the crosslinked polymer network after the Ca^2+^ ionic crosslinking reaction together with the carbonization process at a temperature higher than 500 °C [38].

According to Tudorachi et al. [39], the maximum degradation temperature, T_max_, is the temperature where the degradation rate is maximum. The DTG thermogram of OPEFB cellulose showed the highest T_max_ at 356.66 °C compared to CMC at 281.66 °C and CMC hydrogel at 293.66 °C. This was due to the high crystalline nature and well-ordered structure of the OPEFB cellulose backbone, which made it more thermally stable than modified cellulose. This statement is supported by the CrI percentage in the XRD analysis. The T_max_ of CMC was the lowest because the modification affected the molecular structure and bonding energy, which caused the CMC to become thermally unstable. El-Sakhawy et al. [40], in their thermal study of CMC and CMC acetate, also found that CMC shifted to a lower temperature due to the reaction with NaOH during the carboxymethylation process, which increases the amorphous structure of CMC. The DTG thermogram shows that the T_max_ of CMC hydrogel was slightly higher than CMC. This might be due to the inclusion of Ca^2+^ ions into the CMC structure through ionic crosslinking, which made the CMC hydrogel backbone more stable. Seki et al. [41], in their study on different crosslinking reagents of CMC/HEC hydrogel, stated that the crosslinked CMC hydrogel has better T_max_ than CMC, which delayed thermal decomposition. From the TGA and DTG thermograms, it can be summarized that CMC hydrogel has a slightly better thermal stability in comparison with CMC.

#### 3.2.4. Field Emission Scanning Electron Microscopy (FESEM)

**Figure 12:**
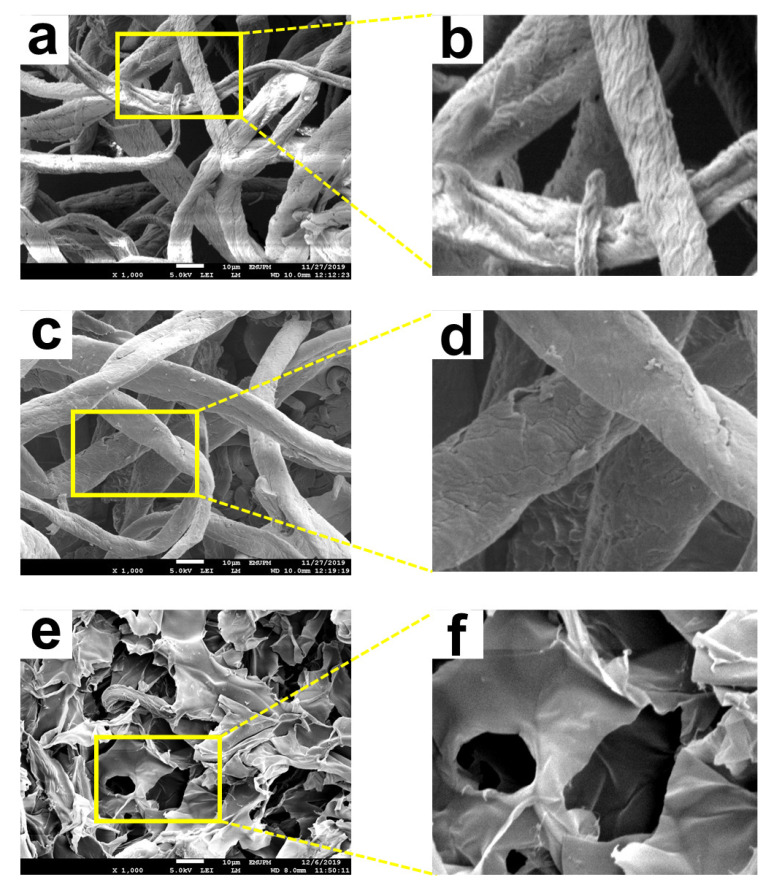
FESEM micrographs of OPEFB cellulose (**a**,**b**), CMC (**c**,**d**), and CMC hydrogel (**e**,**f**) at 1000×.

FESEM is a type of electron microscope used to observe extremely fine morphology features on the surface of a whole or fractioned subject. A high-resolution sample image can be monitored and focused in high magnification using FESEM. Figure 12a–f show the images of OPEFB cellulose, CMC, and CMC hydrogel.

Figure 12a,b show the surface morphology of OPEFB cellulose, which reveals the rough surface of cellulose in fiber form. It is found that OPEFB cellulose fibers were twisted and ruptured. The rough string-like form of OPEFB cellulose fibers may be due to the use of strong chemicals and a high temperature during the cellulose extraction process [42]. Sreekala et al. [43] also stated that mercerization treatment during cellulose extraction to remove natural and artificial impurities such as hemicellulose, lignin, wax, and pectin produced a rough surface topology. Figure 12c,d show the FESEM micrographs of CMC. The CMC micrographs reveal that the surface of the modified cellulose had become less rough and wrinkled. In addition, the string-like OPEFB cellulose agglomerated and attached. The results obtained are consistent with a study by Muhamad Parid et al. [42], which stated that the decrease in roughness is due to the modification by the etherifying agent that reduced the crystalline nature of cellulose. Figure 12e,f show morphological images of freeze-dried CMC hydrogel. The CMC hydrogel has irregular sizes of macropores. According to Rahman et al. [44], the difference in pore size in hydrogel could be due to different crosslinking densities, which can lead to substantially lower or higher water uptake before freeze-drying. The majority of the CMC interfiber macropores were connected, which was desirable for water entrapment because diffusion and the presence of the pores enhanced the swelling ratio of the hydrogel.

## 4. Conclusions

In this study, macroporous CMC hydrogel was successfully synthesized and optimized from OPEFB cellulose through the OVAT method. The optimum gel content was 27.61% with 47.37 g/g degree of swelling. The best optimization conditions to produce CMC hydrogel with an optimum percentage of gel content were at 20% (*w*/*v*) of CMC, 1% (*w*/*v*) of CaCl_2_, 24 h reaction time, and ambient temperature. The modification of OPEFB cellulose via carboxymethylation and ionic crosslinking can convert the abundantly available biomass waste from the oil palm industry and can be further utilized as a biodegradable sorbent material and drug carrier in various applications. 

## Figures and Tables

**Table 1:** Crystallinity index of OPEFB cellulose, CMC, and CMC hydrogel.

Sample	Crystallinity Index (%)
OPEFB cellulose	48.70
CMC	32.77
CMC hydrogel	4.24

## Data Availability

Data sharing not applicable.
